# Genomic Characterization of the Historical Smallpox Vaccine Strain Wyeth Isolated from a 1971 Seed Vial

**DOI:** 10.3390/v15010083

**Published:** 2022-12-28

**Authors:** Nádia Vaez G. Cruz, Matheus Nobrega Luques, Terezinha Marta Pereira P. Castiñeiras, Orlando Costa Ferreira Jr, Regina Helena S. Peralta, Luciana J. da Costa, Clarissa R. Damaso

**Affiliations:** 1 Instituto de Biologia do Exército, Rio de Janeiro 20911-270, Brazil; 2 Instituto de Biofísica Carlos Chagas Filho, Universidade Federal do Rio de Janeiro, Rio de Janeiro 21941-902, Brazil; 3 Núcleo de Enfrentamento e Estudo de Doenças Infecciosas Emergentes e Reemergentes (NEEDIER), Universidade Federal do Rio de Janeiro, Rio de Janeiro 21941-902, Brazil; 4 Departamento de Genética, Instituto de Biologia, Universidade Federal do Rio de Janeiro, Rio de Janeiro 21941-902, Brazil; 5 Faculdade de Medicina, Universidade Federal Fluminense, Niterói 24220-000, Brazil; 6 Instituto de Microbiologia Paulo de Góes, Universidade Federal do Rio de Janeiro, Rio de Janeiro 21941-902, Brazil

**Keywords:** smallpox vaccine, vaccinia virus, monkeypox virus, poxvirus

## Abstract

The Wyeth strain of vaccinia virus (VACV) produced by Wyeth Pharmaceuticals was supposedly used to manufacture the old freeze-dried American smallpox vaccine, Dryvax, until its discontinuation in 2008. Although the genomic sequences of numerous Dryvax clones have been reported, data on VACV-Wyeth genomes are still lacking. Genomic analysis of old VACV strains is relevant to understand the evolutionary relationships of smallpox vaccines, particularly with the recent resumption of smallpox vaccination in certain population groups as an attempt to control the worldwide monkeypox outbreak. Here we analyzed the complete genome sequences of three VACV-Wyeth clonal isolates obtained from a single seed vial donated to the Brazilian eradication program in the 1970s. Wyeth clones show >99.3% similarity to each other and >95.3% similarity with Dryvax clones, mapping together in clade I of the vaccinia group. Although the patterns of SNPs and INDELs comparing Dryvax and Wyeth clones are overall uniform, important differences were detected particularly at the ends of the genome. In addition, we detected recombinant events of clone Wyeth A111 and the Dryvax clone Acam2000, suggesting that other regions of the genomes may have similar patchy patterns of recombination. A small-scale serological survey using VACV-Wyeth as antigen in ELISA assays revealed that 63 of the 65 individuals born before the end of smallpox vaccination in Brazil still have anti-VACV IgG antibodies, demonstrating the usefulness of the VACV-Wyeth strain in future extended serological studies of the Brazilian population.

## 1. Introduction

In 1980, WHO declared the eradication of smallpox after implementing an intensified eradication program, which achieved high levels of vaccination coverage worldwide and widespread surveillance. The WHO program also contributed to the expansion of national vaccination programs and to the robust production of the smallpox vaccine by several countries [1].

Different strains of vaccinia virus (VACV; genus *Orthopoxvirus*, family *Poxviridae*) were used as the smallpox vaccine during the eradication era. The strains differed in reactogenicity and immunogenicity and were used in the production mostly of calf-based and egg-based vaccines [1,2,3]. Countries were free to choose the VACV strains used in their national vaccination program. However, two main strains, Lister and the New York City Board of Health (NYCBH), were strongly recommended by the WHO as an attempt to standardize the production protocols and achieve the desired levels of potency and quality control during the eradication campaign [1,4].

Dryvax was an American calf-based freeze-dried smallpox vaccine produced by Wyeth Pharmaceuticals, Philadelphia, PA, USA, allegedly using the Wyeth strain. It was licensed from 1943 to 2008 and was considered of lower pathogenicity than the Lister-based vaccines [1,5]. VACV strain Wyeth was supposedly derived from the NYCBH strain [6], but a detailed historical investigation on the origins of the vaccine produced by Wyeth Pharmaceuticals suggested that, in fact, it may represent a pool of different VACV strains, including the historical Beaugency lymph [7]. Virus stocks were likely progressively mixed during the numerous merger processes of different American vaccine manufacturers, which have been part of Wyeth Pharmaceuticals’ history since the late 19th century [7,8].

In Brazil, the Oswaldo Cruz Institute in Rio de Janeiro was the main manufacturer of smallpox vaccines distributed nationwide as part of the systematic vaccination program [9,10]. Calf-based vaccines were preferentially used and were manufactured with VACV strain IOC, which probably derived from the Beaugency lymph brought to Brazil from France in the late 19th century and was exclusively produced in Brazil. The other three production centers in Brazil used different VACV strains [1,10]. On some occasions, seed stocks of Wyeth-based smallpox vaccine were supplied to the Brazilian program by Wyeth Pharmaceuticals to overcome temporary supply shortages in the 1970s (Wilson RJ to Henderson DA, Sanofi Pasteur archives, 8 September 1970, personal communication). However, there is little information on whether Wyeth vaccine seed was in fact used to produce smallpox vaccines in Brazil, the range of distribution and if the seed virus was the same as the one used for manufacturing the Dryvax vaccine licensed in the USA.

Advances in massive sequencing technologies over the past decade have allowed for a better understanding of the genetic relationships between different smallpox vaccines [11,12,13,14,15,16,17,18,19,20,21]. In this context, the complete genomic sequencing of Lister, IOC and Dryvax vaccine strains showed high genetic diversity among clonal isolates obtained from a single vaccine vial, especially in the genome ends where virulence genes are located [12,17,20,21]. Such studies are particularly important for the selection of less reactogenic but equally immunogenic clonal isolates than primary vaccine stocks. For example, the current American second-generation vaccine ACAM2000 is a clonal isolate of the formerly used first-generation Dryvax vaccine [22,23]. Interestingly, although VACV-Wyeth is supposedly the original strain used to manufacture the Dryvax vaccine, its full genome sequence has never been reported.

Since May 2022, the world scenario has changed because of the multi-country outbreak of Mpox [24,25]. Smallpox vaccines protect against Mpox, but vaccine stocks are limited worldwide. This fact exposes the need to broaden vaccine options against Mpox and to invest in improved second- and third-generation vaccine candidates. For that, it is essential to improve the knowledge on the phylogenetic relationships and genome sequence of different VACV strains.

Therefore, given the historical importance of the strain VACV-Wyeth during the smallpox eradication era, we sequenced and analyzed the complete genomes of three clonal isolates of VACV-Wyeth produced by Wyeth Laboratories in 1971 and stored as original seed vials until the present days.

## 2. Materials and Methods

### 2.1. Viruses and Clone Isolation

Original ampoules containing lyophilized VACV-Wyeth grown in LLC-MK2 cells were manufactured by Wyeth Pharmaceuticals on 1/11/1971 and donated to Instituto Oswaldo Cruz, Rio de Janeiro, RJ, during the smallpox eradication campaign. The ampoules were kindly provided to our laboratory by Herman Schatzmayr (Instituto Oswaldo Cruz) in 1981. Ampoules were reconstituted in PBS and serially diluted in saline to inoculate BSC-40 cells with 10^−3^ dilution (MOI of 0.0001) for 72 hpi under semi-solid medium. Isolated viral plaques with representative plaque size were selected randomly and submitted to two additional rounds of plaque purification to obtain three clones denominated A111, A211, and A311 [12,26]. For stock amplification and virus purification, VACV-Wyeth clones were propagated in BSC-40 cells at an MOI of 0.01 for 48 h. Infected cells (post-nuclear extracts) were then sedimented through to 36% sucrose cushions, and the resulting pellets were submitted to ultracentrifugation through a 25% to 45% potassium tartrate gradient, as described [12,26].

### 2.2. Genome Sequencing, Assembly, and Annotation

Genomic DNA from purified particles of VACV-Wyeth clones was isolated using Wizard^®^ Genomic DNA Purification Kit (Promega, Madison, WI, USA) and used for library preparation using the Illumina genomic Nano kit. The libraries were submitted to high-throughput 2 × 125 + 8 paired-ended sequencing using Illumina HiSeq 2500. Following quality control, Illumina raw reads were used for de novo assembly using Velvet version 1.2.10 and the resulting contigs were re-assembled using SeqMan (DNAStar package; Lasergene Inc., Madison, WI, USA) for proper positioning of the inverted terminal repeats (ITRs). Assemblies were curated by mapping the raw reads to each final contig by using BWA version 0.7.12 [27], followed by visualization in Tablet vs. 1.13.07_21. Genomes were annotated based on the sequences of VACV-IOC clone B141 and Dryvax clone DPP16 using GATU software [28], followed by manual editing using CLC Main Workbench vs. 7.0. GenBank accession numbers of the genome sequences of VACV-Wyeth clones A111, A211, and A311 are OP751801, OP751802, and OP751803, respectively.

### 2.3. Genome Analyses

Genomes were aligned by using Mafft version. 7.0 with default parameters. The full alignments were used in Base-by-base for the graphical presentation of substitutions, insertions, and deletions distributed along the genomes [29]. Alignments spanning the conserved region between orthologs of VACV-Cop F9L and A24R were also used to construct a maximum likelihood tree using MEGA X version 10.2.6, opting for the General Time Reversible (GTR) model, as suggested by the Model Test tool, NJ/BioNJ input tree, five-category discrete gamma distributed with invariant site, and 1000 bootstrap replicates [30]. To generate a median-joining network (MJN), the full genome alignment was first edited to remove columns with >99% nucleotide similarity by using Jalview version 2.11.1.1, followed by gap removal using Bioedit (version 7.2.5). As a result, all columns in the final alignment contained at least one SNP. This final alignment was used in Network vs. 5.0.1.1 to generate the MJN using default parameters (https://www.fluxus-engineering.com/ (accessed on 4 September 2022)).

A full genome alignment of the three Wyeth clones and Dryvax clones was used to in Simplot to generate a similarity profile plot with parameters indicated in the Figure legend [31]. Different regions of the multi-alignment were extracted by using Geneious Prime version 2021.2.2 and used in the Bootscan tool of Simplot to generate recombination plots, using parameters specified in the Figure legend.

In addition to the genomes of Wyeth clones A111, A211, and A311 (Genbank access numbers indicated above), the following genome sequences were used in the genome analyses described in this manuscript: Genbank accession numbers are cowpox virus strains Austria 1999 (HQ407377.1), HumBer07/1 (KC813509.1), Germany 1990 (HQ420896), Brighton Red (AF482758), horsepox virus strain MNR76 (DQ792504); Mulford_1902 (MF477237); vaccinia virus strains: IOC_B141 (KT184690), IOC_B388 (KT184691), Cantagalo (CTGV)_CM01 (KT013210), CTGV_ALE-H2 (MW018156), CTGV_CG-04 (MW018155), CTGV_MI-233 (MW018153), CTGV_VI-04 (MW018154), Serro-2 (KF179385), WR (AY243312), Copenhagen (M35027), IHDW (KJ125439), Lister 107 (DQ121394), Lister LC16m8 (AY678275); Dryvax clones DPP11 (JN654978), DPP13 ((JN654980), DPP15 (JN654981), DPP16 (JN654982), DPP17 (JN654983), DPP20 (JN654985), DPP25 (KJ125438), ACAM2000 (AY313847), Tiantan clones TP3 (KC207810), and TP5 (KC207811), Tashkent clones TKT3 (KM044309) and TKT4 (KM044310); Ectromelia virus strain Moscow (AF012825.2); camelpox strain CMS (AY009089); and variola virus Bangladesh 1975 (DQ437581).

### 2.4. Study Population and Serum Samples

A panel of 130 serum samples was obtained from volunteers, 65 of whom were born between 1944 and 1977, while the other 65 participants were born between 1981 and 1996. All participants signed a free and informed consent form. Inclusion criteria were whether participants were over 18 years of age, resided in Brazil during childhood (up to 10 years), and were not revaccinated or had accidental vaccinia virus exposure after ending smallpox vaccination in Brazil in 1979. As a positive control, we used a pool of convalescent sera from two laboratory workers accidently infected with vaccinia virus strains WR and IOC and the serum from a laboratory worker recently vaccinated with the ACAM2000 smallpox vaccine (with intervals of 10 years between boosters). Blood samples were collected via venipuncture and subjected to low-speed centrifugation to obtain the serum fraction. Serum aliquots were inactivated at 56 °C for 30 min and stored at −80 °C.

### 2.5. Indirect ELISA (Enzyme Linked Immunosorbent Assay)

Purified particles of VACV-Wyeth clone A111 were inactivated by exposure to UV light (A_254nm_) for 10 min. Samples (1 μg/mL) in bicarbonate/carbonate coating buffer (pH 9.6) were used to coat microtiter 96-well ELISA plates (Nunc MaxiSorp^®^, Thermo Fisher Scientific, São Paulo, Brazil) at 4 °C overnight. After washing with PBS containing 0.3% Tween20 (PBST), plates were blocked with 5% nonfat dry milk in PBST at 37 °C for 1 h, washed again, and incubated in triplicates with sera diluted 1:100 at 37 °C for 30 min. HRP-conjugated anti-human IgG antibody diluted 1:10,000 was added at 37 °C for 30 min, followed by the addition of 3,3′,5,5′-tetramethylbenzidine (TMB—Sureblue™; KPL) at room temperature for 20 min. The reaction was stopped by adding 0.1 N HCl. Absorbance values were determined at 450 nm using SpectraMax M5e (Molecular Devices, San Jose, CA, USA). Cut-off limits (99.7% CI) were calculated using the mean OD_450nm_ value (0.158) obtained for sera from 66 individuals born after 1979 plus three standard deviations (3SD = 0.192) for the upper limit (0.351) and plus two standard deviations (2SD = 0.124) for the lower limit (0.282). The upper limit was set as 1 Arbitrary Unit (AU). Sera above 1 AU were considered positive and sera below 0.80 AU were considered negative [32].

### 2.6. Ethical Considerations

This descriptive, cross-sectional study was part of two projects reviewed and approved by the Research Ethics Committee of the Clementino Fraga Filho University Hospital of the Federal University of Rio de Janeiro, under the protocol numbers CAAE 36631714.0.0000.5257 and 62281722.5.0000.5257.

### 2.7. Statistical Analyses

All statistical analyses were performed using GraphPad Prism^®^ vs. 8.02 (GraphPad Software, San Diego, CA, USA). To compare viral plaque areas, we used the one-way ANOVA with the Tukey’s multiple comparisons test. Vaccinated and unvaccinated groups were first tested for normal distribution using the Shapiro–Wilk Normality test, followed by a two-tailed unpaired t test and Mann–Whitney U test.

## 3. Results

### 3.1. Clonal Isolation and Genomic Characterization of VACV-Wyeth

**Figure 1:**
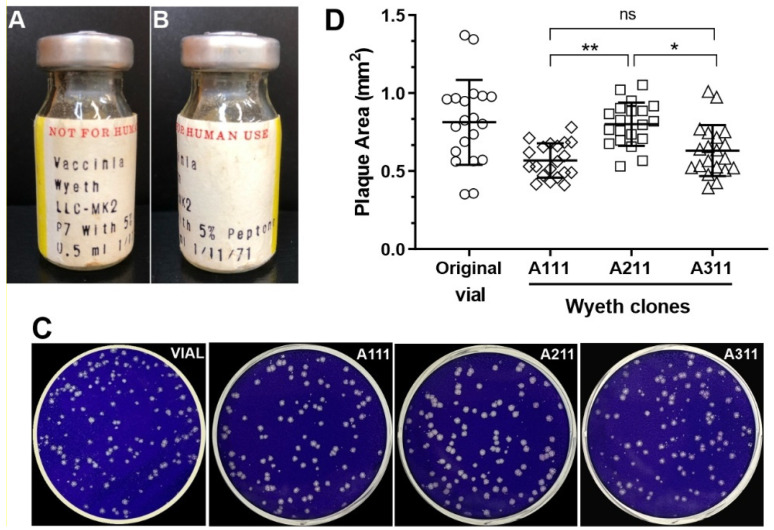
Clone isolation of VACV-Wyeth and plaque phenotype. (**A**,**B**) Original vial of lyophilized VACV-Wyeth propagated in LLC-MK2 in 1971. (**C**) Representative images of crystal-violet stained BSC-40 cells infected with 10^−6^ dilution of the original vial before plaque selection and stocks of Wyeth clones A111, A211, and A311. (**D**) Random viral plaques (*n* = 20) were photographed at 4× magnification, and the individual areas were measured. Circles: plaques of the original vaccine vial; diamonds: plaques of clone A111; squares: plaques of clone A211; triangles: plaques of clone A311. Asterisks: * *p* ≤ 0.05 and ** *p* ≤ 0.01; ns means non-significant.

To determine the genome sequence of VACV strain Wyeth, we reconstituted an original vial of lyophilized VACV-Wyeth propagated in monkey LLC-MK2 cells in 1971 (Figure 1A,B) and inoculated endpoint dilutions onto BSC-40 cells monolayers. Three isolated and size-representative plaques were collected randomly and used in two additional rounds of clonal selection (Figure 1C). Clones A111 and A311 had similar plaques size whereas clone A211 produced plaques nearly 1.3× larger than the other clones (Figure 1D). All three clones were propagated in cell cultures and purified by ultracentrifugation. DNA isolated from purified virus particles were used for Illumina sequencing.

The sequencing statistics are described in Table 1. The genomes of the three clones have approximately 200 kb each, although the genome of clone A111 is slightly larger than the two other genomes because of extended sequencing of the non-coding regions in the genome ends. For all clones, the ITRs are more than 16-kb long with 28 ORFs duplicated in each ITR for clone A111 and A211, and 27 ORFs for clone A311, which lacks one fragment of the ortholog of the 77 kDa cowpox host-range gene in both ends. A total of 249 ORFs were annotated for clones A111 and A211, and 246 ORFs were detected in the genome of clone A311. The major ORF differences between the three clones are described in Table S1. Worth noting clone A111 has an intact ortholog of VACV_Cop-M1L, while clones A211 and A311 have truncated genes. M1L encodes an apoptosis inhibitor that targets the apoptosome. It is absent from MVA strain and truncated in some Dryvax clones [20,33]. On the other hand, clone A111 has a fragmented ortholog of VACV_Cop-E5R, while clones A211 and A311 have intact copies. E5R encodes a major component of the virosomes and is also truncated in ACAM2000 and other Dryvax clones [20]. Mutations described in the literature as associated with altering VACV plaque phenotype were not found in the genomes of Wyeth clones.

**Figure 2:**
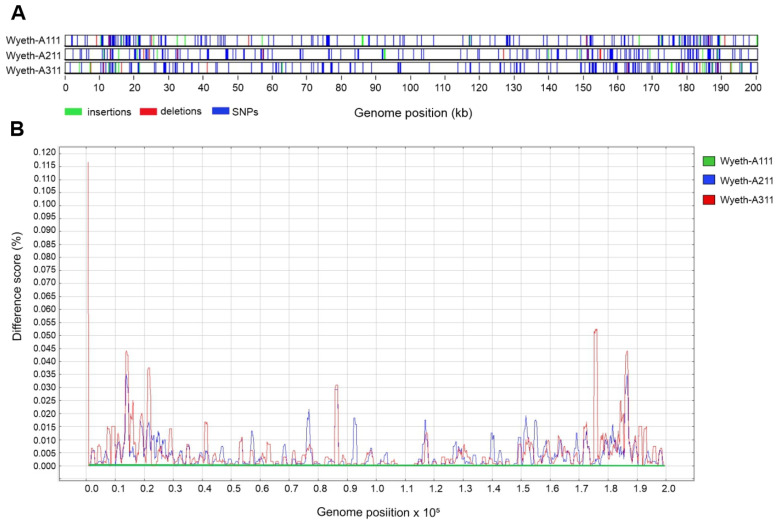
Genome-wide analysis of differences comparing the genomes of VACV-Wyeth clones A111, A211, and A311. The full-genome alignment of VACV-Wyeth clones A111, A211, and A311 was analyzed using Base-by-base [29]. (**A**) A graphical presentation of sequence variations per site (SNPs and INDELs) was revealed by pairwise comparison of the three Wyeth genomes. (**B**) Differences per site detected in the genomes of clones A211 (red line) and A311 (blue line) in relation to clone A111 (green bottom line) were scored along the genome.

The identity scores shown in Table 1 support the strong relatedness of the clonal isolates, as expected, but also reveal differences within the three Wyeth genomes. To analyze such differences, we compared all three genomes simultaneously and screened for the genome-wide distribution of single nucleotide polymorphisms (SNPs), insertion and deletions (INDELs) (Figure 2A). The base-by-base analysis shows a prevalence of SNPs over INDELs in all three clones, and a higher accumulation of differences near the genome ends (variable region) when compared with the central genome, which is expected based on the conservation of the central region. The differences were scored and plotted along the genomes. Overall, clone A311 accumulates higher difference scores in relation to clone A111 than clone A211 does (Figure 2B), which is in accordance with the identity scores reported in Table 1. Again, a higher density of alterations was observed in the genome ends than in the central region (Figure 2B).

### 3.2. Phylogenetic Analysis of VACV-Wyeth Clones and the Relationship with Dryvax Strain

**Figure 3:**
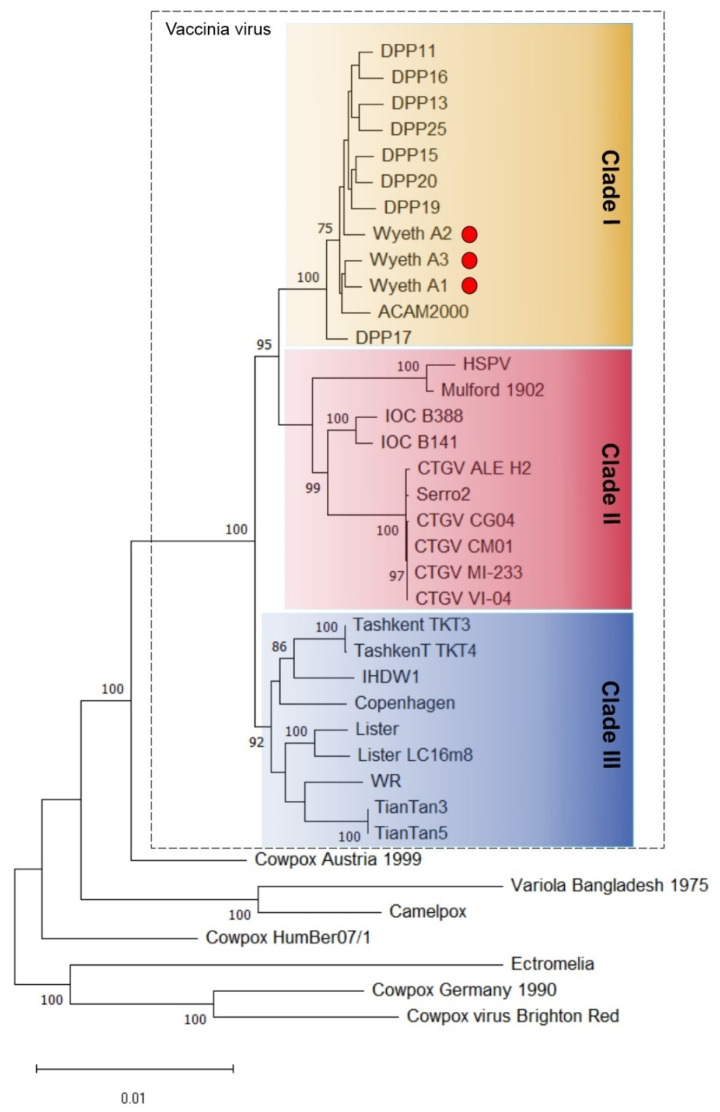
Phylogenetic reconstruction of VACV-Wyeth clones A111, A211, and A311. A multialignment of the conserved region of 38 orthopoxvirus genomes was used to infer a maximum likelihood tree using MEGA X, opting for the General Time Reversible (GTR) model and five-category discrete gamma distributed with invariant site. Numbers next to branch nodes indicate the percentage of 1000 replicates of bootstrap support (values >70 are shown). The scale bar indicates the number of substitutions per site. Colored boxes indicate the three main clades in the vaccinia group. The Wyeth clones are indicated with red circles.

The phylogenetic analysis based on the central conserved region of several orthopoxvirus genomes is shown in Figure 3 and reveals the regular topology of three clades in the VACV group, i.e., clade I (former American/Dryvax clade), clade II (former horsepox/Brazilian clade), and clade III (former Eurasian clade) [12,26]. The three clones of VACV-Wyeth were placed within the clade I, together with clones of the Dryvax vaccine (Figure 3). This topology is in accordance with the high similarity levels observed among the genomes of Wyeth and Dryvax clones, which vary from 95.31% Dryvax_DPP17 to 99.11% with ACAM2000. This range of genome similarity is also observed between Dryvax clones, suggesting that Wyeth and Dryvax could be considered a single strain.

**Figure 4:**
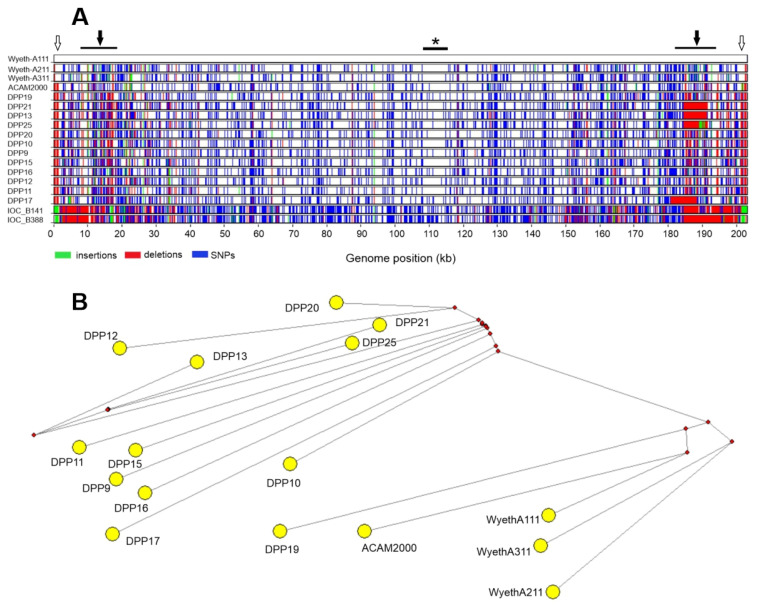
Genome-wide distribution of differences and Median-joining network analysis comparing the genomes of clones of VACV-Wyeth and Dryvax. (**A**) A multialignment of the full genomes of VACV-Wyeth and Dryvax clones (clade I) was analyzed by using Base-by-base to generate a graphical map of genetic differences (insertions, SNPs, and deletions) in relation to the genome of VACV-Wyeth A111 used as reference. VACV-IOC clones B141 and B388 (clade II) were also included for sake of comparison. Asterisk indicates a highly conserved region of the genomes. Black arrows indicate regions in the genomes of Dryvax clones enriched in deletions in relation to Wyeth A111. Open arrows indicate deletions unique to the genome ends of the Dryvax clones. (**B**) Median-joining network analysis using a Jalview-edited multialignment of the full genomes of VACV-Wyeth and Dryvax clones. Branch lengths are proportional to the number of accumulated SNPs. The small red nodes (median vectors) indicate unsampled or extinct common ancestors.

Because variants of VACV-Wyeth and Dryvax group together in clade I, we included Dryvax genomes into a comparative genome-wide map of differences (Figure 4A). The base-by-base analysis revealed highly conserved regions between Dryvax and Wyeth clones, e.g., a region that expands from the orthologs of VACV-Cop D8L through A2L (Figure 4A, top black bar with asterisk). However, important differences were noted in the genome ends, represented by several small and a few large deletions in the Dryvax genomes, not detected in the Wyeth clones (Figure 4A, top black bars with black arrows). These deletions occur in the left and right junctions of the ITRs and variable regions and have been thoroughly described previously [20]. We also observed small deletions found only in the non-coding regions of the ITRs of Dryvax clones, which correspond to small insertions in all three Wyeth clones (Figure 4A, open arrows). These insertions correspond to four repeats of 14, 19, 25 and 30 nucleotides, which, although exclusive to Wyeth clones, are inserted into a region of multiple repeats shared by the Dryvax and Wyeth clones (Figure S1, asterisks). Although we could detect some differences in the map of SNPs and INDELs for members of clade I, the pattern could be considered overall uniform when compared with the clearly contrasting map observed for VACV-IOC genomes, which belong to clade II, a sister clade to clade I (Figure 4A).

To investigate the relationship between clones of Dryvax and Wyeth in more details, we focused on a SNP-based distance analysis. The median-joining network shown in Figure 4B reveals a segregation between both groups. Overall, the Dryvax clones cluster separately from the three VACV-Wyeth clones, except for clone DPP19 and ACAM2000. In fact, the latter shares an unsampled or extinct ancestor with Wyeth clone A111. Clones A211 and A311 are more related to each other than to clone A111, in accordance with the identity scores shown in Table 1. Dryvax clones appear to have accumulated more genetic diversity than Wyeth clones based on the longer branches, although more clonal isolates of the Wyeth strain need to be sampled and analyzed to support this conclusion.

### 3.3. Recombination Analysis of VACV-Wyeth and Dryvax Clones

Recombination is one of the major drivers in the evolution of poxviruses. Recombination in microregions is particularly interesting and have been described as occurring intraspecies and intrastrain, particularly in smallpox vaccine strains, generating a patchy pattern suggestive of traces of past recombination events [12,20,34]. Low bootstrap values between variants within the same clade are generally considered to be indicative of such events [35,36].

**Figure 5:**
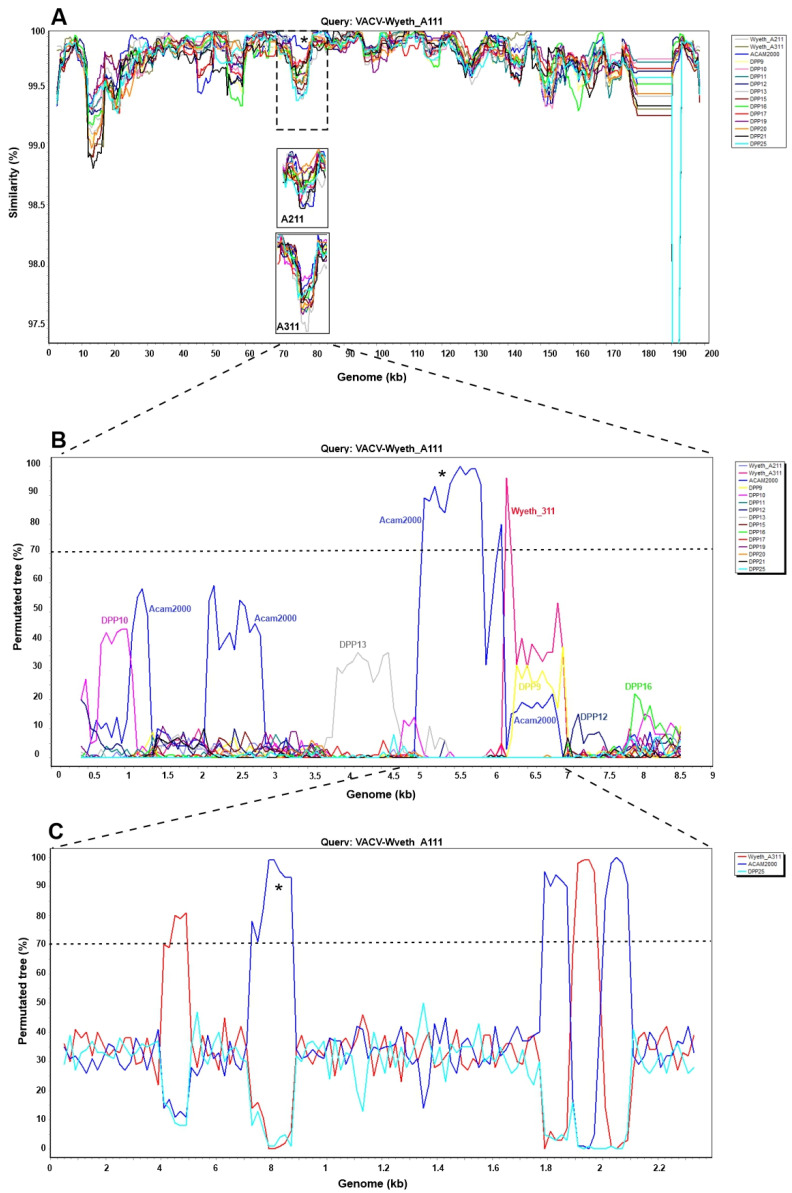
Similarity profile and Bootscan analysis to detect recombination in the genomes of VACV-Wyeth clones. (**A**) Similarity plot of the whole-genome multi-alignment using Simplot, opting for VACV-Wyeth A111 sequence as query, window size 5000, step size 500. The asterisk and the dotted box indicate the region of lower similarity of Wyeth A111 with the Wyeth clones A211 and A311, and the Dryvax clones, except for Acam2000. (**B**) A 15-kb region spanning from 70 kb to 85 kb of the multi-alignment shown in A was analyzed by Bootscan, opting for Wyeth A111 as query, window size 800, and step size 70. (**C**) Two putative recombinant events with Acam2000 and Wyeth 311 were identified in (**B**), and the corresponding 2.5-kb region was extracted and reanalyzed by Bootscan, opting for Wyeth A111 as query, window size 100, and step size 20, and using Acam2000 and Wyeth 311 as donor sequences, and DPP25 as non-donor strain.

Recombination analysis in large genomes such as poxvirus genomes, requires detailed studies. Therefore, as a first general analysis to map more prominent recombination events between VACV-Wyeth clones and Dryvax clones we used Simplot to map similarity across the entire genome and identify contrasting regions of major and minor similarity. As show in Figure 5A, the overall pattern of similarity is uniform and minor SNP differences highlighted in Figure 4 were occluded by the window parameters chosen for this genome-wide analysis. However, a region around 75 kb of the alignment stood out as all Dryvax clones, but ACAM2000 clearly reduced the level of similarity with Wyeth A111 (Figure 5A, asterisk). This was not observed when Wyeth A211 and Wyeth 311 were used as query (Figure 5A, insets).

To explore the possibility of recombination, we bootscanned the region spanning 70 kb to 85 kb using several Dryvax clones, Wyeth 211, and Wyeth 311 as possible sequence donors (Figure 5B). Several peaks suggestive of recombination events were detected, but most of them below a robust boostrap threshold of 70%. However, two peaks stood out in the region of greatest similarity with Acam2000 shown in Figure 5A (dotted box with asterisk), suggesting recombination events with Acam2000 and Wyeth 311.

To confirm these recombination events in the Wyeth A111 genome, we bootscanned this 2.5 kb-long microregion using the classic approach of selecting two possible sequence donors (Acam2000 and Wyeth 311) and DPP25 as the non-donor strain, as it showed the lowest similarity value for this region in Figure 5A. The bootscan plot in Figure 5C strongly suggests the occurrence of five recombination events, as shown by the peaks with robust bootstrap support. These findings support the close relationship of Wyeth A111 and Acam2000 suggested by the MJN analysis (Figure 4B) and indicate that more detailed recombination analysis is needed in other microregions of the genome.

### 3.4. Serological Response to VACV-Wyeth of Individuals Vaccinated against Smallpox in Childhood

The ongoing Mpox outbreak has motivated the search for novel vaccines against orthopoxviruses around the world and has stimulated the conduction of clinical studies to evaluate protection against Mpox after smallpox vaccination and serosurvey studies to establish the immunological status of individuals, particularly healthcare workers, previously vaccinated against smallpox in childhood [37,38,39,40].

In Brazil, the systematic vaccination campaign against smallpox was carried out by the national eradication program until 1975, four years after the last smallpox case in the country. The systematic campaign consisted, among other actions, of bringing the population together for an annual day of mass vaccination, in order to guarantee high vaccination and revaccination coverage. After 1975, the vaccine was still available in some primary health centers and hospitals until 1979, but it was not mandatory. In this context, we implemented a small-scale serosurvey study to investigate serological status of individuals vaccinated against smallpox during childhood in Brazil. Our goal was also to investigate the performance of VACV-Wyeth as antigen for ELISA tests as this strain has advantages over the Brazilian strains VACV-IOC and VACV-Cantagalo due to the generation of high titers and low PFU:particle ratio, which will be important in future studies.

**Figure 6:**
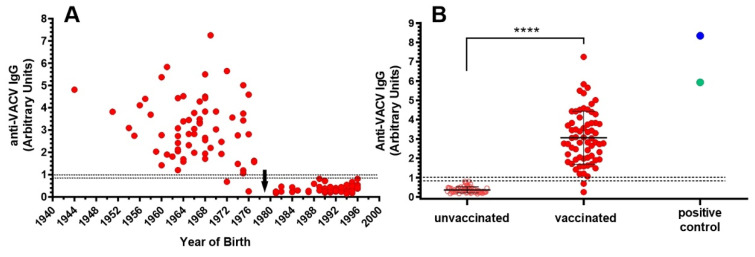
Detection of vaccinia virus IgG antibodies in serum samples of 130 individuals born before and after the completion of smallpox vaccination in Brazil. Detection was performed by ELISA using purified inactivated VACV-Wyeth clone A111 as antigen. Results for each serum are represented by individual dots and expressed as arbitrary units. (**A**) Distribution of anti-VACV IgG values according to the year of birth. Black arrow points to 1979, when smallpox vaccination was ended in Brazil. (**B**) Serological response to VACV-Wyeth was plotted according to the presumed vaccination status. Individuals born before 1979 were considered vaccinated and individuals born after 1979 were considered unvaccinated. The blue circle refers to a laboratory worker recently and repeatedly vaccinated with the smallpox vaccine. The green circle corresponds to a pool of two convalescent sera from laboratory workers accidently infected with VACV. Dotted lines indicate the upper and lower limits of the test cut-off. **** *p* < 0.0001 (Mann–Whitney).

Sera from 65 individuals born before 1979 and 65 individuals born after 1979 were screened for the presence of anti-VACV IgG using inactivated Wyeth A111 clone as the antigen to coat ELISA plates. Figure 6A shows the distribution of anti-VACV IgG levels in the study population. Although older individuals received more vaccine booster doses due to annual mass vaccination days, the decline in their IgG levels is greater than in younger individuals. Therefore, the highest levels of IgG were not necessarily found in the oldest individuals (Figure 6A). Individuals born before 1979 were presumably vaccinated and, accordingly, all but two were positive, i.e., they had antibodies that recognized the VACV-Wyeth antigens. On the other hand, all individuals born after 1979 were likely not vaccinated, and as expected were negative (Figure 6B).

## 4. Discussion

An important feature of orthopoxviruses is the well-known cross-immunity shown by species of the genus [41]. In fact, this feature was essential for the eradication of smallpox in the late 1970s, as the vaccine used against smallpox, a disease caused by the variola virus, contained another orthopoxvirus, vaccinia virus [1,8]. First-generation vaccines used 40 years ago do not meet current safety, potency and tolerance standards required by regulatory bodies. Therefore, second and third generation vaccines have been developed over the years, but to a limited extent because the main purpose has been to increase the vaccine stockpile of smallpox biodefense programs in some countries [42,43]. In this context, the monkeypox outbreak in 2022 surprised the world as the number of cases increased exponentially worldwide and exposed the urgent need to expand our vaccine options against orthopoxvirus infection [25].

Despite being the first vaccine ever generated in the world, the history of the smallpox vaccine is still full of mysteries, and little is known about the evolutionary relationships of the various VACV strains used as the smallpox vaccine [7,11,15,19,26]. Here we analyzed the genetic makeup of three clones isolated from a VACV-Wyeth seed sample produced in 1971. The three clones map within clade I of the vaccinia lineage together with all Dryvax clones. This clade was previously known as the American/Dryvax clade. However, evidence accumulating over the past decade shows that some American VACV strains such as WR and IHDW, while supposedly derived from the NYCBH strain, do not cluster with Dryvax clones, but rather map within the former Eurasian clade, now referred to as clade III [11,12,20]. Therefore, the old clade names no longer seemed appropriate.

The former American Dryvax vaccine was produced by Wyeth Pharmaceuticals, likely using the Wyeth strain, which in turn allegedly derived from the NYCBH strain [1,5,6]. However, there are many unknowns in the early history of the smallpox vaccine industry when using mixed seed strains to produce smallpox vaccines was not unusual [7,44]. Wyeth Pharmaceuticals, for example, has gone through several mergers with other vaccine producers and the Dryvax licensed in 1943 may not have necessarily been manufactured using the same VACV seed strain until the final production in the early 1980s [7]. Even the New York City Health Department produced vaccines in calves in 1871 by mixing the Beaugency lymph and a vaccine strain brought from Cuba. On the other hand, vaccines passed only in human arms used a strain obtained from England in 1856 [7]. Therefore, the potential mixed nature of seed strains used by Wyeth Pharmaceuticals associated with the continuous passage in calves throughout the years have certainly increased the genetic diversity of Dryvax, as observed by previous works [11,20,23]. However, we show here that all three Wyeth clones are closely related to Dryvax clones, particularly Wyeth clone A111, which is close to the currently American vaccine ACAM2000. The high genetic similarity of Dryvax and Wyeth suggests that they represent a single strain and support the historical origin link between them.

Several studies have shown that clonal isolates of smallpox vaccines, including Dryvax, accumulate large deletions, gene translocations and duplications in both genome ends, with higher frequency in the 3′ junction of the ITR with the variable region [11,12,20]. However, our data show that SNPs are the major source of variations between the Wyeth clones without major deletions when compared with Dryvax genomes. In this context, the Wyeth genomes are larger than the Dryvax genomes which vary from 194.4 kb to 198.8 kb, except for ACAM2000 which has 199.2 kb, reinforcing the increased gene loss in Dryvax clones than in Wyeth genomes. Because the 1971 vial of VACV-Wyeth was a seed strain propagated in cell culture and not in calves like the Dryvax vaccine, it may have been subjected to a better control of passage number or stocks may have been produced from a recently cloned viral isolate. However, investigation of other clones is needed to help us clarify these issues.

In addition to SNPs, poxvirus evolution also involves recombination, and such events in microregions are particularly relevant to better understand the evolutionary relationships of close members within VACV clades [35,36]. We performed a general recombination analysis that highlighted a region of Wyeth A111 genome that likely recombined with Acam2000 (or a Acam2000-like sequence) and Wyeth 311 as past event in the evolutionary history of the Wyeth strain. These data agree with the Median-Joining network analysis that clusters Wyeth A111 and Acam2000 and encourages future in-depth recombination analysis of other regions of the genomes.

As mentioned earlier, the smallpox vaccine is known to protect against Mpox based on laboratory evidence and animal studies [45,46]. However, evidence of its effectiveness against human Mpox is still limited and, to date, indicate levels of protection ranging from 80 to 87% [47]. In this context, not only clinical trials are underway to investigate vaccine effectiveness worldwide, but also large-scale surveillance studies to investigate the serological status of health workers previously vaccinated against smallpox in childhood, as well as seroconversion after MPXV infection in different population groups [37,38,39,48]. For the latter, the use of VACV as the source of antigen for ELISA tests is an interesting option to avoid the use of purified MPXV, which requires a higher level of biosafety when compared with VACV. Here, we show that the use of VACV-Wyeth as antigen is a good option to investigate the serological status of healthy individuals previously vaccinated against smallpox in childhood. Although other VACV strains should work well in general, sensitivity can vary depending on the strain [38], requiring further comparative investigation with other VACV strains. An advantage of VACV-Wyeth is that it propagates at higher titers than other strains available in Brazil, such as VACV-IOC, used in the 1970s as a smallpox vaccine, or the VACV-Cantagalo field strain. It also generates lower PFU:virus particle ratio, which is important for future dual ELISA and neutralization paired tests. In the present small-scale study, most individuals supposedly vaccinated in childhood and born from 1944 to 1977 have antibodies against VACV. Two individuals born in 1972 and 1976 were negative, probably because they were not vaccinated even though vaccination was still available until 1979 in some health centers and hospitals.

This small-scale study is in line with a similar study recently conducted in the Netherlands [38] and highlights the importance of serological surveys to assess prior humoral immunity of a given population to orthopoxviruses. Previous history of smallpox vaccine in childhood does not necessarily correlate with high levels of anti-VACV IgG. This is relevant in the current context when different population groups are recommended to receive the smallpox vaccine as pre-exposure or post-exposure prophylaxis against Mpox and especially in a scenario of scarce vaccine supply [25].

## Figures and Tables

**Table 1:** Genomic features of the genomes of VACV-Wyeth clones A111, A211, and A311.

	VACV-Wyeth Clones		
	A111	A211	A311
Genome size (bp)	200,123	199,816	199,892
ITR size (pb)	16,945	16,117	16,128
Number of reads	3,716,924	3,295,152	3,816,236
% mapped reads	94.34%	92.45%	93.26%
Genome coverage (x)	2184.38	1852.41	2168.59
Number of annotated ORFs	249	249	246
% Identity in relation to			
A111	100	99.45	99.38
A211	99.45	100	99.55
A311	99.38	99.55	100

## Data Availability

The data analyzed in this study are included within the paper.

## Supplementary Materials

The following supporting information can be downloaded at: https://www.mdpi.com/article/10.3390/v15010083/s1, Table S1: Major ORF differences between Wyeth clones A211 and A311 in relation with clone A111. Figure S1: Alignment of the left genome end of Wyeth and Dryvax clones.
