# Supplementary material for: Genomic Characterization of the Historical Smallpox Vaccine Strain Wyeth Isolated from a 1971 Seed Vial
Source: Viruses. 2022 Dec 28;15(1):83. doi: 10.3390/v15010083 (PMC9864299; doi:10.3390/v15010083)
Supplement: Supplementary file 1 [file viruses-15-00083-s001.zip › Figure S1.pdf]

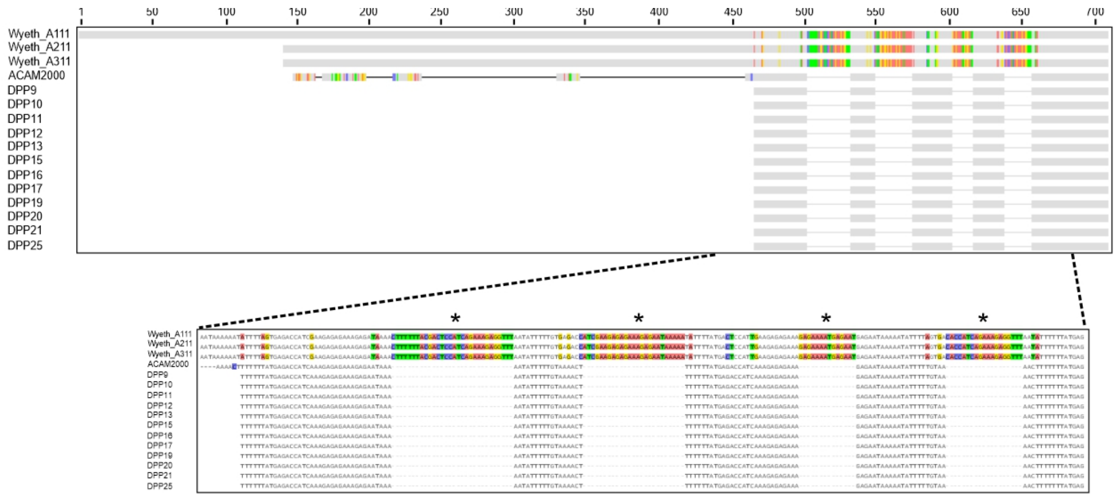

Figure S1. Insertions in the genome ends of VACV-Wyeth clones. The left end of a multialignment of Dryvax and VACV-Wyeth genomes was analyzed in Geneious Prime 2022.2.2. Four insertions present only in VACV-Wyeth genomes are highlighted in color (top panel) and indicated with asterisks in the bottom panel, which shows a zoomed-in inspection of the region.
