# Supplementary material for: Genomic Characterization of the Historical Smallpox Vaccine Strain Wyeth Isolated from a 1971 Seed Vial
Source: Viruses. 2022 Dec 28;15(1):83. doi: 10.3390/v15010083 (PMC9864299; doi:10.3390/v15010083)
Supplement: Supplementary file 1 [file viruses-15-00083-s001.zip › Table S1.pdf]

Table S1. Major ORF differences between Wyeth clones A211 and A311 in relation to clone A111

Wyeth clones	ORFs in Wyeth genome[a]		
	Present[a]	Absent[a]	Product (ortholog of VACV-Cop)
A211	036.1		Fragment of C4L. NFκB inhibitor. Different patterns of fragmentation in A111, A211, and A311
	042.1		Fragment of M1L. Apoptosis inhibitor. Intact ORF in clone A111
		074	Fragment of E5R. Virosome component. Intact ORF in clone A211 and A311, but fragmented in A111
		181	Fragment of A39R. Semaphorin-like protein. Different patterns of fragmentation in A111, A211, and A311
A311		024	Fragment of 77 kDa cowpox host-range gene. Different patterns of fragmentation in A111, A211, and A311.
	042.1		Fragment of M1L. Apoptosis inhibitor. Intact ORF in clone A111
		074	Fragment of E5R. Virosome component. Intact ORF in clone A211 and A311, but fragmented in A111
		181	Fragment of A39R. Semaphorin-like protein. Different patterns of fragmentation in A111, A211, and A311
		228	Fragment of 77 kDa cowpox host-range gene. Different patterns of fragmentation in A111, A211, and A311.

[a]ORF number is indicated.
